# Cardiometabolic Risk Factors in Young Adults Who Were Born Preterm

**DOI:** 10.1093/aje/kwu443

**Published:** 2015-05-05

**Authors:** Marika Sipola-Leppänen, Marja Vääräsmäki, Marjaana Tikanmäki, Hanna-Maria Matinolli, Satu Miettola, Petteri Hovi, Karoliina Wehkalampi, Aimo Ruokonen, Jouko Sundvall, Anneli Pouta, Johan G. Eriksson, Marjo-Riitta Järvelin, Eero Kajantie

**Keywords:** blood pressure, glucose metabolism, hypertension, late preterm, obesity, prematurity

## Abstract

Adults who were born preterm with a very low birth weight have higher blood pressure and impaired glucose regulation later in life compared with those born at term. We investigated cardiometabolic risk factors in young adults who were born at any degree of prematurity in the Preterm Birth and Early Life Programming of Adult Health and Disease (ESTER) Study, a population-based cohort study of individuals born in 1985–1989 in Northern Finland. In 2009–2011, 3 groups underwent clinical examination: 134 participants born at less than 34 gestational weeks (early preterm), 242 born at 34–36 weeks (late preterm), and 344 born at 37 weeks or later (controls). Compared with controls, adults who were born preterm had higher body fat percentages (after adjustment for sex, age, and cohort (1985–1986 or 1987–1989), for those born early preterm, difference = 6.2%, 95% confidence interval (CI): 0.4, 13.2; for those born late preterm, difference = 8.0%, 95% CI: 2.4, 13.8), waist circumferences, blood pressure (for those born early preterm, difference = 3.0 mm Hg, 95% CI: 0.9, 5.1; for those born late preterm, difference = 1.7, 95% CI: −0.1, 3.4), plasma uric acid levels (for those born early preterm, difference = 20.1%, 95% CI: 7.9, 32.3; for those born late preterm, difference = 20.2%, 95% CI: 10.7, 30.5), alanine aminotransferase levels, and aspartate transaminase levels. They were also more likely to have metabolic syndrome (for those born early preterm, odds ratio = 3.7, 95% CI: 1.6, 8.2; for those born late preterm, odds ratio = 2.5, 95% CI: 1.2, 5.3). Elevated levels of conventional and emerging risk factors suggest a higher risk of cardiometabolic disease later in life. These risk factors are also present in the large group of adults born late preterm.

Each year, 14.9 million infants worldwide, approximately 1 of every 9 who are liveborn, are born before 37 weeks of gestation (1–3). There is increasing evidence that the smallest and most immature of them, such as those born with very low birth weight (<1,500 g) or born very preterm (<32 weeks), have higher levels of cardiometabolic risk factors as adults, including elevated blood pressure (4–9), impaired glucose regulation (10, 11), and atherogenic lipid profiles (9). However, most of this evidence is limited to these conventional cardiometabolic risk factors in the extreme groups of adults born at very low birth weight or very preterm.

Of all preterm infants in the United States, 70% are born late preterm, that is, between 34 and 36 weeks of gestation (12); in the European Union, more than 80% of preterm infants are born moderately preterm, that is, between 32 and 36 weeks of gestation (2). Yet, only few studies investigating adult cardiometabolic risks have included the whole range of preterm births. Results from these studies have suggested that a linear relationship exists between a shorter gestation period and higher blood pressure in adult life (13, 14). Should a similar “dose-response” relationship exist for other cardiometabolic risk factors, even moderately higher risks in the much larger group of people born late or moderately preterm could potentially cause a larger public health burden.

We hypothesized that preterm birth at all levels is associated with cardiometabolic risk factors in adult life. We tested this hypothesis in a cohort of young adults by using both conventional risk factors, such as body size and the established components and criteria of the metabolic syndrome, and emerging risk factors that may reflect specific pathophysiological pathways, such as body composition, plasma apolipoproteins, uric acid, and markers of inflammation and fatty liver disease.

## METHODS

**Figure 1.**
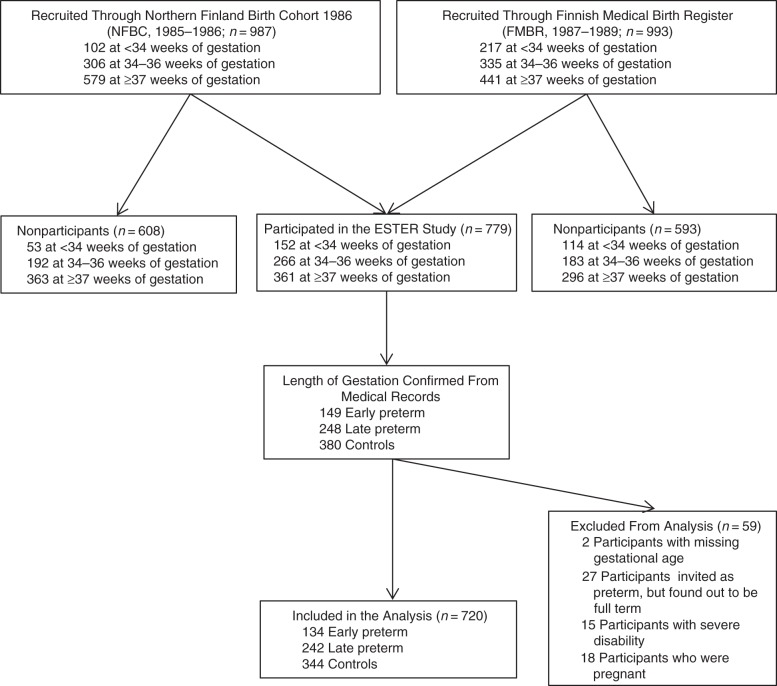
Flow chart of the study population, Northern Finland, 2009–2011. All subjects who were recruited into the study were born in Northern Finland in 1986–1989. In the Finnish Medical Birth Register data, there were 83 individuals in the random control group who had missing gestational age, 58 of whom did not participate in the Preterm Birth and Early Life Programming of Adult Health and Disease (ESTER) Study and 25 of whom participated. Ten subjects who were born early preterm, 1 subject who was born late preterm, and 4 controls reported severe disability and were excluded from the analysis. Five subjects who were born early preterm, 5 subjects who were born late preterm, and 8 controls reported being pregnant and were excluded from the analysis. Three of the excluded subjects had more than 1 reason for exclusion.

The present study is part of the Preterm Birth and Early Life Programming of Adult Health and Disease (ESTER) Study, the design of which is depicted in Figure 1. The original cohort comprised 1980 individuals born in Northern Finland, 987 (49.8%) of whom came from the Northern Finland Birth Cohort 1986 (NFBC; born in 1985–1986) (13); the remaining 993 (50.2%) were recruited from all individuals born in the same geographical area in 1987–1989 and were identified through the Finnish Medical Birth Register (FMBR). The numbers of those who were invited and those who participated are shown in Figure 1. From the NFBC, we invited all individuals who were born either early preterm (born before 34 gestational weeks) or late preterm (born at 34–36 gestational weeks); from the FMBR, we recruited all individuals who were born early preterm. Furthermore, to attain approximately double the total number of participants in the late preterm group as in the early preterm group, we recruited a randomly selected sample of individuals born late preterm from the FMBR. From both cohorts, we recruited a group of randomly selected controls (Figure 1). Because of slight variation in number of individuals recruited through the cohorts, we adjusted for recruitment cohort (NFBC or FMBR) in all the analyses.

In 2009–2011, a total of 779 individuals participated in a clinical study. After verification of the length of gestation from medical records (15), we excluded 2 participants whose length of gestation could not be verified and 27 subjects thought to be born preterm who were actually born at term (Web Table 1 available at http://aje.oxfordjournals.org/). Those who were pregnant (*n* = 18) or who reported having cerebral palsy (*n* = 6), a mental disability (*n* = 7), and/or another severe physical disability (*n* = 3) were excluded from analysis because these conditions might affect the main outcomes. Three subjects had more than 1 criterion for exclusion. In addition, 7 subjects reported the use of β-blockers for indications other than hypertension, and they were excluded from the blood pressure analysis. Thus, there were 134 participants who were born early preterm, 242 participants who were born late preterm (16), and 344 controls who were born at term. The perinatal data are shown in Table 1.

**Table 1.** Perinatal, Neonatal, and Current Characteristics of Cases and Controls, Northern Finland, 2009–2011

Characteristic	Early Preterm (*n* = 134)				*P* Value^a^	Late Preterm (*n* = 242)				*P* Value^a^	Controls (*n* = 344)			
	No.	%	Mean (SD)	No. Missing		No.	%	Mean (SD)	No. Missing		No.	%	Mean (SD)	No. Missing
Male	65	48.5			0.95	120	49.6			0.86	168	48.8		
Peri- and neonatal characteristics														
Pregnancy with multiple fetuses	32	23.9			<0.001	34	14.0			<0.001	4	1.2		
Maternal hypertension^b^	17	12.7		0	0.51	36	15.2		5	0.10	36	10.6		4
Maternal preeclampsia^c^	32	23.9		0	<0.001	22	9.3		5	<0.001	16	4.7		4
Maternal gestational diabetes	4	3.5		21	0.28	11	5.0		24	0.03	6	1.8		9^d^
Maternal smoking during pregnancy	21	16.5		7	0.94	46	19.3		4	0.44	57	16.8		5
Cesarean delivery	80	59.7			<0.001	67	27.7			<0.001	41	11.9		
Gestational age, weeks			31.8 (2.0)		<0.001			35.8 (0.8)		<0.001			40.1 (1.2)	
Birth weight, grams			1,786 (493)		<0.001			2,674 (515)		<0.001			3,576 (483)	
Birth weight SD score			−0.73 (1.4)		<0.001			−0.63 (1.3)		<0.001			−0.02 (1.0)	
Small for gestational age	22	16.4			<0.001	30	12.4			<0.001	7	2.0		
Large for gestational age	4	3.0			0.91	5	2.1			0.41	11	3.2		
Respirator treatment		68	50.7			<0.001	28	11.6			<0.001	2	0.6	
Duration of respirator treatment, days^e^			7.0 (10.4)		0.55			2.8 (2.7)		0.86			2.5 (0.7)	
Supplementary oxygen	98	73.1			<0.001	93	38.4			<0.001	7	2.0		
Duration of supplementary oxygen, days^f^			9.0 (14.1)		0.15			2.0 (3.6)		0.59			1.3 (1.9)	
Current														
Age, years			23.1 (1.4)		<0.001			23.2 (1.3)		<0.001			23.6 (1.1)	
Parental educational level				1	0.51				5	0.62				4
Basic or less or unknown	12	9.0				19	8.0				20	5.9		
Secondary	81	60.9				134	56.5				207	60.9		
Lower-level tertiary	13	9.8				31	13.1				45	13.2		
Upper-level tertiary	27	20.3				53	22.4				68	20.0		
Maternal history of hypertension	19	15.1		8	0.53	39	16.9		3	0.18	42	12.8		17
Maternal history of diabetes	3	2.4		8	0.41	17	7.4		12	0.08	13	4.0		16
Maternal history of myocardial infarction or stroke	2	1.6		7	0.13	4	1.7		12	0.08	1	0.3		17
Paternal history of hypertension	19	15.2		9	0.62	39	17.3		16	0.97	56	17.1		17
Paternal history of diabetes	6	4.8		9	0.07	20	8.8		16	0.63	33	10.1		17
Paternal history of myocardial infarction or stroke	8	6.4		9	0.81	15	6.6		15	0.69	19	5.8		17
Self-reported physical activity, MET-hours/week			23.5 (13.8)	4	0.08			24.9 (14.5)	7	0.35			26.0 (13.8)	10
Daily smoking	39	29.1			0.08	54	22.3			0.82	74	21.5		
Amount of alcohol doses per week				3	0.89				9	0.31				12
0	23	17.6				51	21.9				57	17.2		
1–3	60	45.8				87	37.3				142	42.8		
4–9	24	18.3				57	24.5				71	21.4		
10 or more	24	18.3				38	16.3				62	18.7		
Use of β-blockers	1	1.4			0.56	2	1.6			0.52	4	2.3		
Hormonal contraception (% of women)	24	34.8			0.30	45	36.9			0.37	74	42.0		

Abbreviations: MET, metabolic equivalent; SD, standard deviation.

^a^ *P* values refer to comparisons between subjects who were born preterm and controls, using Student's *t* test or Pearson's χ^2^ test (and Fisher's exact test for use of antihypertensive drug).

^b^ Gestational or chronic hypertension.

^c^ Includes superimposed preeclampsia.

^d^ Subjects who had missing data on maternal gestational diabetes included subjects whose mothers did not undergo an oral glucose tolerance test despite risk factors and thus have uncertain gestational diabetes status.

^e^ Among those treated in respirator.

^f^ Among those treated with supplementary oxygen.

We compared persons who participated with those who did not or who were excluded from the analysis because of pregnancy or physical disability. This comparison was based on perinatal data from the NFBC database (participants from NFBC) and the FMBR (participants born between 1987 and 1989). In addition, we compared the results of the clinical examinations that took place at 16 years of age among those NFBC members who had participated in that examination. The comparative data are shown in Web Table 2.

### Perinatal data

The perinatal data from the participants recruited through the NFBC (born in 1985–1986) came from the cohort database; the information was originally collected from medical records (13). We collected corresponding data from those invited through the FMBR (born in 1987–1989) from their hospital and maternal welfare clinic records. We retrospectively confirmed the length of gestation (determined by ultrasonography in 62.7% of preterm infants and 53.1% of controls) (15) and diagnoses of maternal gestational diabetes, hypertension (gestational or chronic), or preeclampsia (including superimposed) according to prevailing criteria by reviewing original hospital records (17, 18). We calculated birth weight standard deviation scores according to Finnish birth weight standards, which are based on 75,061 singletons born in 1979–1983 in Finland (19). Although these standards are based on newborn measurements, they assume a constant coefficient of variation (standard deviation/mean ratio) at each gestational week, analogous to fetal growth standards based on ultrasound measurements (20, 21). We defined “small for gestational age” as less than 2 standard deviations below and “large for gestational age” as more than 2 standard deviations above the mean for sex and length of gestation.

### Clinical examination

The subjects attended the clinic at 7:30–9:00 am after an overnight fast and were examined by 2 trained study nurses. After a 5-minute rest in a sitting position, participants had their blood pressure measured 3 times from the right upper arm using an automatic oscillometric blood pressure monitor (Omron M10-IT Intellisense, Omron Healthcare Co., Kyoto, Japan). Subjects with a systolic blood pressure of 140 mm Hg or higher or a diastolic blood pressure of 90 mm Hg or higher were classified as having hypertension (22). Height was measured 3 times. The waist circumference, which was measured midway between the lowest rib and the iliac crest, and the hip circumference, which was measured at the widest point of the hip, were both measured twice. The mean values of these measurements, the waist-hip ratio, and body mass index (BMI; weight in kilograms divided by height in meters squared) were calculated. Subjects with a BMI higher than 30 were classified as obese. We used a segmental multifrequency bioelectrical impedance (InBody 3.0, Biospace Co., Seoul, Korea) to assess body composition (weight, lean body mass, fat mass, and percentage of body fat). Blood samples were collected after fasting and 2 hours after a 75-gram oral glucose load. The laboratory analyses are described in the Web Appendix. To test for metabolic syndrome, we used the criteria from the joint interim statement by Alberti et al. (23). Three or more of the following 5 criteria had to be met: 1) central obesity (waist circumference ≥94 cm in men and ≥80 cm in women); 2) triglycerides ≥1.7 mmol/L; 3) high density lipoprotein cholesterol (HDL-C) level <1.03 mmol/L in men and <1.29 mmol in women; 4) blood pressure ≥130/85 mm Hg; and 5) fasting plasma glucose level ≥5.6 mmol/L or type 2 diabetes mellitus. We calculated the fatty liver index as (e^0.953 × ln (triglycerides, mg/dL) + 0.139 × BMI + 0.718 × ln (γ glutamate, U/L) + 0.053 × waist circumference− 15.745^)/(1 + e^0.953 × ln (triglycerides, mg/dL) + 0.139 × BMI + 0.718 × ln (γ glutamate, U/L) + 0.053 × waist circumference − 15.745^) × 100 (conversion for triglycerides: 1 mg/dL = 18.0182 × 1 mmol/L) (24). The participants completed questionnaires that included questions about their medical histories, medication use, socioeconomic status, and lifestyles. Socioeconomic status was assessed using the highest educational level of each subject's more highly educated parent and was categorized in 4 levels (dummy coded). Self-reported physical activity levels were converted to total metabolic equivalent hours per week (25). The research protocol was approved by the Coordinating Ethics Committee at Helsinki and Uusimaa Hospital District, and all the participants provided written informed consent.

### Statistical methods

All statistical analyses were performed using SPSS for Windows, version 21 (SPSS Inc., Chicago, Illinois). The main outcome variables according to pathophysiological pathways are presented in the Web Table 3. To account for non-normal distribution of residuals or heteroscedasticity of the dependent variable, we log-transformed all continuous variables except blood pressure, height, and concentrations of uric acid, albumin, and urea. We compared the characteristics of the subjects in the early preterm and late preterm groups with those of the controls using Student's *t* test and the χ^2^ test and compared the main outcomes using linear or logistic regression. We first estimated the crude effect of preterm birth in model 1, which included age, cohort (NFBC or FMBR), and sex. In model 2, to assess the total effect, we adjusted for variables in model 1 in addition to parental and prenatal confounding factors (including parental educational level as a proxy of childhood socioeconomic position, maternal smoking during pregnancy, and birth weight standard deviation scores as indicators of fetal conditions during pregnancy) and, for analyses of dichotomous and biochemical outcomes, parental history of hypertension, diabetes, and myocardial infarction or stroke as proxies for genetic susceptibility. We report additional adjustments for maternal hypertension in pregnancy and gestational diabetes in the Results. In model 3, we adjusted for the variables in model 1 and for the current characteristics at the time of study: height (for body composition and blood pressure) or BMI (for blood pressure and biochemical risk factors), physical activity level, and smoking status. For fatty liver index, we also adjusted for current alcohol use. Model 4 included both the confounders and intermediate factors to assess the direct association of preterm birth with these outcomes. The associations between preterm birth and the outcomes were similar in men and women, except for HDL-C and apolipoprotein A1, for which the *P* values for interaction terms between sex and early preterm birth were 0.035 and 0.019 (adjusted for age and sex), respectively. For this reason, we report comparisons of plasma lipid levels separately for women and men. All *P* values are 2-sided.

## RESULTS

### Obesity and body composition

**Figure 2.**
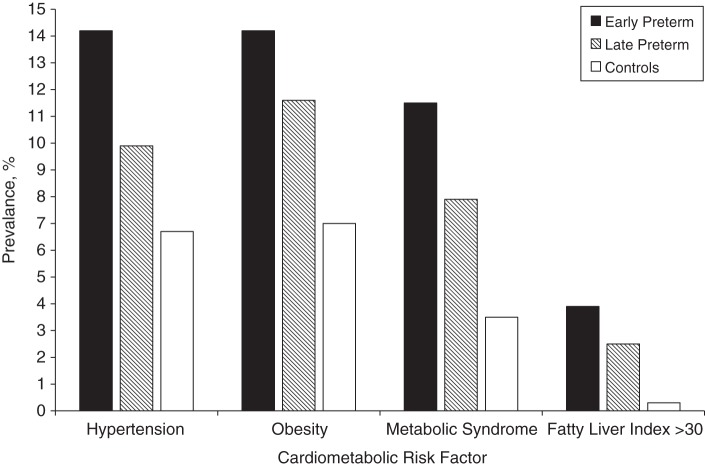
Prevalence of hypertension, obesity, metabolic syndrome, and fatty liver index greater than 30 in adults who were born early preterm or late preterm compared with adults born at term (controls), Northern Finland, 2009–2011.

The characteristics of the study groups are presented in Table 1. Subjects who were born preterm were approximately 2 times more likely to be obese than were controls (Figure 2 and Table 2), which was reflected in their higher mean body mass indices and waist circumferences (Table 3). Waist circumferences, waist-hip ratios, and (with borderline significance for the early preterm group) percentages of body fat were higher in both early and late preterm groups than in the controls; lean body masses were similar (Table 3).

**Table 2.** Odds Ratios for Hypertension, Obesity, Fatty Liver Index, and Metabolic Syndrome in Adults Who Were Born Preterm Compared With Controls Who Were Born at Full Term, Northern Finland, 2009–2011

Outcome Variable and Model^a^	Early Preterm			Late Preterm			Total No.
	OR	95% CI	*P* Value	OR	95% CI	*P* Value	
Obesity							
1	2.2	1.1, 4.1	0.02	1.7	1.0, 3.1	0.06	720
2	2.5	1.2, 4.8	0.009	1.8	1.0, 3.3	0.06	704
3	2.0	1.0, 4.0	0.04	1.8	1.0, 3.3	0.05	699
4	2.4	1.2, 4.9	0.01	1.9	1.0, 3.5	0.05	683
Hypertension							
1	2.7	1.3, 5.3	0.005	1.7	0.9, 3.2	0.10	711
2	3.0	1.4, 6.2	0.004	1.6	0.8, 3.2	0.16	695
3	2.6	1.3, 5.4	0.01	1.6	0.8, 3.1	0.15	690
4	2.4	1.1, 5.3	0.03	1.4	0.7, 2.9	0.37	674
Metabolic syndrome							
1	3.7	1.6, 8.2	0.002	2.5	1.2, 5.3	0.02	711
2	4.3	1.9, 10.1	<0.001	2.4	1.1, 5.4	0.03	696
3	3.6	1.6, 8.3	0.003	2.7	1.2, 5.8	0.01	692
4	4.6	1.9, 11.1	<0.001	2.7	1.2, 6.0	0.02	677
Fatty liver index >30							
1	13.6	1.5, 120.0	0.02	8.6	1.0, 72.8	0.05	706
2	11.6	1.3, 106.1	0.03	7.5	0.8, 65.4	0.07	691
3	12.5	1.4, 111.2	0.02	8.5	1.0, 72.0	0.05	681
4	10.8	1.2, 100.2	0.04	7.0	0.8, 61.4	0.08	666

^a^ Model 1 was adjusted for sex, age, and cohort. Model 2 was adjusted for the variables in model 1 and parental educational level, maternal smoking during pregnancy, birth weight standard deviation score, and parental hypertension, diabetes, and myocardial infarction/stroke. Model 3 was adjusted for the variables in model 1 and self-reported physical activity level and daily smoking (and fatty liver index for alcohol user). Model 4 was adjusted for the variables in models 2 and 3.

**Table 3.** Geometric Mean Values^a^ of Body Size and Composition Variables in Controls and Mean Differences Between Young Adults Who Were Born Preterm and Controls, Northern Finland, 2009–2011

Measure and Model^b^	Controls, mean (SD)		Early Preterm			Late Preterm			Total No.
	Women	Men	Mean Difference, %	95% CI	*P* Value^c^	Mean Difference, %	95% CI	*P* Value^c^	
Height,^d^ cm	164.0 (6.0)	177.6 (7.0)							
1			0.4	−0.9, 1.6	0.59	0.5	−0.6, 1.5	0.38	720
2			1.7	0.5, 3.0	0.006	1.7	0.7, 2.7	0.001	704
3			0.3	−1.0, 1.6	0.64	0.4	−0.7, 1.4	0.49	699
4			2.0	0.8, 3.2	0.001	1.8	0.8, 2.8	<0.001	683
Body mass index	22.0 (0.19)	23.0 (0.19)							
1			1.8	−1.8, 5.3	0.29	2.9	0.1, 5.8	0.03	720
2			2.6	−0.9, 6.3	0.15	3.7	0.8, 6.7	0.007	704
3			1.9	−1.5, 5.5	0.27	3.1	0.2, 6.0	0.04	699
4			3.4	−0.6, 6.8	0.98	4.1	1.1, 7.1	0.004	683
Waist circumference, cm	73.7 (0.13)	82.3 (0.10)							
1			3.6	1.2, 6.1	0.002	3.3	1.3, 5.3	0.001	719
2			4.4	1.9, 7.0	<0.001	3.9	1.9, 6.0	<0.001	703
3			3.5	1.0, 6.0	0.007	3.2	1.2, 5.3	0.002	698
4			4.0	1.4, 6.6	0.002	3.6	1.8, 5.7	<0.001	682
Waist-hip ratio	0.81 (0.05)	0.90 (0.05)							
1			1.8	0.7, 2.9	<0.001	1.3	0.4, 2.1	0.003	719
2			1.8	0.8, 3.0	<0.001	1.3	0.5, 2.2	0.003	703
3			1.6	0.5, 2.7	0.003	1.3	0.4, 2.1	0.004	698
4			1.7	0.6, 2.9	0.03	1.4	0.5, 2.3	0.002	682
Lean body mass, kg	43.2 (0.13)	61.5 (0.15)							
1			−0.2	−2.8, 2.5	0.88	1.3	−0.9, 3.5	0.30	718
2			2.5	−0.2, 5.2	0.07	3.6	1.4, 5.8	0.001	702
3			−0.5	−2.5, 1.5	0.61	0.8	−0.8, 2.5	0.32	697
4			0.4	−1.6, 2.6	0.68	1.6	−0.1, 3.3	0.07	681
Fat mass, kg	15.7 (0.52)	11.5 (0.57)							
1			8.5	−0.1, 18.9	0.08	11.7	3.7, 20.4	0.004	718
2			10.1	0.1, 21.1	0.05	13.5	5.1, 22.6	0.001	702
3			9.1	−0.5, 19.7	0.06	11.8	3.7, 20.6	0.004	697
4			11.4	1.1, 22.7	0.03	14.1	5.5, 23.4	<0.001	681
Percentage body fat, %	26.0 (0.32)	15.4 (0.42)							
1			6.2	−0.4, 13.2	0.07	8.0	2.4, 13.8	0.004	718
2			5.2	−1.6, 12.5	0.14	7.4	1.7, 13.4	0.01	702
3			7.0	0.6, 14.0	0.03	8.5	3.1, 14.2	0.002	697
4			8.1	1.2, 15.5	0.02	9.7	4.0, 15.7	<0.001	681

Abbreviations: CI, confidence interval; SD, standard deviation.

^a^ The geometric mean is the *n*th root of the product of *n* values. The geometric SD corresponds to the percentage of the increase in a variable that corresponds to a 1-SD change in the logarithm of the variable.

^b^ Model 1 was adjusted for sex, age, and cohort. Model 2 was adjusted for the variables in model 1 and parental educational level, maternal smoking during pregnancy, and birth weight SD score. Model 3 was adjusted for the variables in model 1 and height (except for height), self-reported physical activity level, and daily smoking. Model 4 was adjusted for the variables in models 2 and 3.

^c^ *P* values are for the differences between means in the preterm groups and controls.

^d^ Values are expressed as arithmetic mean (SD) in centimeters.

### Blood pressure

Hypertension was 2 to 3 times more common in adults who were born preterm (Figure 2 and Table 2), although this was not statistically significant for the late preterm group. Adults who were born early preterm had systolic blood pressures that were 3.0 mm Hg higher and diastolic blood pressures that were 2.6 mm Hg higher than did the controls (Table 4). The difference remained statistically significant after adjustment for covariates.

**Table 4.** Geometric Mean Values^a^ of Blood Pressure and Biochemical Measurements in Controls and Mean Differences Between Adults Who Were Born Preterm and Controls, Northern Finland, 2009–2011

Measure and Model^b^	Controls, mean (SD)	Early Preterm			Late Preterm			Total No.
		Mean Difference	95% CI	*P* Value^c^	Mean Difference	95% CI	*P* Value^c^	
Systolic blood pressure,^d^ mm Hg	116.3 (12.3)							
1		3.0	0.9, 5.1	0.005	1.7	−0.1, 3.4	0.06	711
2		3.2	1.1, 5.4	0.003	1.5	−0.3, 3.3	0.10	695
3		2.7	0.7, 4.8	0.01	1.4	−0.3, 3.1	0.11	699
4		2.1	−0.0, 4.	0.05	0.8	−1.0, 2.5	0.42	674
Diastolic blood pressure,^d^ mm Hg	75.4 (7.6)							
1		2.6	0.9, 4.2	0.002	1.2	−0.1, 2.5	0.07	711
2		2.3	0.7, 4.0	0.006	0.8	−0.5, 2.1	0.24	695
3		2.4	0.9, 4.0	0.002	1.0	−0.2, 2.3	0.11	699
4		1.7	0.1, 3.3	0.04	0.4	−0.9, 1.7	0.56	674
Fasting plasma glucose level, mmol/L	5.1 (1.09)							
1		0.8	−0.8, 2.4	0.35	0.3	−1.0, 1.6	0.68	710
2		0.8	−0.9, 2.4	0.36	0.0	−1.3, 1.4	0.99	695
3		0.5	−1.1, 2.1	0.53	0.1	−1.2, 1.4	0.84	691
4		0.3	−1.3, 2.0	0.71	−0.3	−1.6, 1.1	0.71	676
2-Hour plasma glucose level, mmol/L	6.6 (1.17)							
1		−0.4	−3.9, 3.2	0.83	1.1	−1.8, 4.0	0.46	695
2		−1.6	−5.2, 2.1	0.40	−0.5	−3.4, 2.5	0.65	680
3		−0.8	−4.1, 2.7	0.66	1.1	−1.7, 4.0	0.44	680
4		−2.1	−5.5, 1.5	0.26	−0.4	−3.3, 2.5	0.91	665
Fasting serum insulin concentration, pmol/L	46.2 (1.51)							
1		8.8	−1.0, 19.6	0.08	10.1	1.9, 19.0	0.02	689
2		7.3	−2.8, 18.4	0.16	7.5	−0.7, 16.5	0.07	674
3		4.0	−4.1, 12.8	0.34	4.8	−4.1, 12.8	0.17	670
4		1.1	−7.1, 10.0	0.80	1.7	−5.0, 8.8	0.64	655
2-Hour serum insulin concentration, pmol/L	150.9 (2.04)							
1		15.5	−0.1, 33.6	0.05	16.5	3.0, 31.7	0.02	656
2		10.4	−4.3, 27.3	0.17	11.7	−0.7, 25.9	0.07	656
3		8.5	−5.8, 25.1	0.26	12.6	0.3, 26.3	0.04	641
4		2.7	−11.2, 18.8	0.71	4.0	−7.5, 16.9	0.51	626
Homeostatic model assessment for insulin resistance	0.90 (1.50)							
1		8.9	−0.9, 19.7	0.08	9.8	1.6, 18.6	0.02	688
2		7.5	−2.6, 18.6	0.15	7.2	−1.0, 16.1	0.09	673
3		4.1	−4.0, 12.9	0.33	4.7	−2.0, 12.9	0.33	669
4		1.2	−7.0, 10.1	0.78	1.5	−5.2, 8.7	0.66	654
Fasting plasma uric acid level,^d^ µmol/L	292.1 (68.8)							
1		20.1	7.9, 32.3	0.001	20.1	10.7, 30.5	<0.001	708
2		19.2	6.4, 31.9	0.003	18.6	8.3, 28.9	<0.001	693
3		20.3	8.7, 32.0	<0.001	18.4	8.9, 27.8	<0.001	689
4		18.8	6.7, 30.9	0.002	16.1	6.3, 25.8	0.001	674
High-sensitivity C-reactive protein level, mg/L	1.0 (3.23)							
1		−3.4	−24.6, 23.7	0.78	−9.4	−25.9, 10.7	0.34	649
2		−5.9	−27.5, 22.2	0.65	−12.2	−28.8, 8.3	0.22	634
3		−11.1	−30.2, 13.2	0.34	−14.0	−29.3, 4.6	0.13	632
4		−17.5	−36.1, 6.4	0.14	−18.8	−33.7, −0.4	0.05	617
Blood leukocyte concentration, 10^9^/L	5.8 (1.29)							
1		5.5	0.0, 11.2	0.05	3.1	−1.2, 7.7	0.16	705
2		4.6	−1.0, 10.5	0.11	2.8	−1.7, 7.5	0.23	690
3		2.9	−2.2, 8.3	0.27	2.0	−2.1, 6.4	0.34	686
4		1.8	−3.5, 7.4	0.50	1.7	−2.6, 6.1	0.45	671
Plasma alanine aminotransferase level, U/L	24.8 (1.67)							
1		9.3	−0.6, 20.1	0.07	15.0	6.4, 24.2	<0.001	710
2		8.2	−2.0, 19.5	0.12	13.5	4.7, 22.9	0.002	695
3		8.4	−1.1, 18.95	0.08	13.3	5.1, 22.1	0.001	685
4		7.6	−2.3, 18.4	0.14	12.1	3.7, 21.1	0.004	670
Plasma aspartate transaminase level, U/L	22.7 (1.41)							
1		5.6	−1.5, 13.2	0.12	11.7	5.5, 18.2	<0.001	708
2		3.7	−3.6, 11.5	0.33	9.9	3.7, 16.6	0.002	693
3		7.4	0.1, 15.1	0.05	12.9	6.6, 19.5	<0.001	683
4		5.5	−1.9, 13.5	0.15	11.2	4.8, 17.9	<0.001	668
Plasma albumin level,^d^ g/L	46.7 (2.6)							
1		0.6	0.08, 1.0	0.02	−0.1	−0.5, 0.3	0.20	710
2		0.4	−0.05, 0.9	0.08	−0.07	−0.5, 0.3	0.36	695
3		0.6	0.1, 1.1	0.01	−0.1	−0.5, 0.3	0.52	691
4		0.5	0.0, 1.0	0.04	−0.1	−0.5, 0.3	0.79	676
Plasma γ glutamate level,^d^ U/L	18.9 (1.77)							
1		3.5	−6.7, 14.8	0.51	5.7	−2.8, 15.0	0.18	710
2		2.1	−8.4, 13.8	0.71	4.1	−4.6, 13.7	0.53	695
3		1.7	−7.9, 12.2	0.74	4.1	−4.0, 12.8	0.33	676
4		−0.4	−10.1, 10.4	0.94	2.4	−5.8, 11.3	0.58	670
Plasma urea level,^d^ mmol/L	5.3 (2.5)							
1		0.3	0.0, 0.5	0.04	−0.01	−0.2, 0.2	0.95	708
2		0.2	−0.1, 0.5	0.13	−0.03	−0.3, 0.2	0.77	693
3		0.3	0.0, 0.6	0.04	0.01	−0.2, 0.2	0.99	689
4		0.2	−0.0, 0.5	0.10	−0.02	−0.3, 0.2	0.83	674

^a^ The geometric mean is the *n*th root of the product of *n* values. The geometric SD corresponds to the percentage of the increase in a variable that corresponds to a 1-SD change in the logarithm of the variable.

^b^ Model 1 was adjusted for sex, age, and cohort. Model 2 was adjusted for the variables in model 1 and parental educational level, maternal smoking during pregnancy, birth weight SD score, and parental hypertension, diabetes, and myocardial infarction or stroke. Model 3 was adjusted for the variables in model 1 and body mass index, self-reported physical activity level, and daily smoking, as well as plasma alanine aminotransferase, aspartate transaminase, and γ glutamate for analyses of alcohol users and height for analyses of mean differences in systolic and diastolic blood pressures. Model 4 was adjusted for the variables in models 2 and 3.

^c^ *P* values are for the differences between means in the preterm groups and controls.

^d^ Values are expressed as arithmetic mean (SD).

### Glucose metabolism

One subject who was born late preterm reported using medication for type 2 diabetes mellitus and was excluded from the analyses of glucose metabolism. Subjects who were born late preterm had higher fasting and 2-hour insulin concentrations and higher indices of homeostatic model assessment for insulin resistance than did the controls (Table 4). These differences were minimally changed after adjustment for parental and prenatal confounders but became attenuated after adjustment for current characteristics. Glucose concentrations were similar in all the groups. Eight (3.3%) of the late preterm subjects and 11 (3.2%) of the controls had impaired glucose tolerance, and 1 (0.4%) subject who was born late preterm was found to have diabetes after taking an oral glucose tolerance test.

### Lipid profile

Women who were born early preterm had 11.4% (95% confidence interval (CI): 5.6, 16.9) lower HDL-C and 9.7% (95% CI: 4.5, 14.7) lower apolipoprotein A1 concentrations than did women in the control group (Web Table 4). The differences remained similar after we controlled for covariates. These associations were not present among men (Web Table 4). There were no differences in triglyceride or total or low-density lipoprotein cholesterol levels. No subjects used lipid-lowering medications.

### Metabolic syndrome

Of the 711 subjects who had adequate data, 46 (6.5%) fulfilled the criteria of metabolic syndrome. Of the controls, 12 (3.5%) had the syndrome. Among those who were born early preterm, 15 (11.5%) had the syndrome (after adjustment for sex, age, and cohort, odds ratio = 3.7, 95% CI: 1.6, 8.2) (Figure 2 and Table 2). Among those who were born late preterm, 19 (17.9%) had the syndrome (odds ratio = 2.5, 95% CI: 1.2, 5.3) (Figure 2 and Table 2). The results were similar when further adjustments were made for covariates (Table 2).

### Other biochemical markers of metabolic syndrome

Subjects who were born preterm were 8 to 13 times more likely to have an intermediate or high fatty liver index (24), which is a proxy of nonalcoholic fatty liver disease (Figure 2, Table 2). Of the individual markers of fatty liver disease, alanine aminotransferase and aspartate transaminase concentrations were higher in those who were born preterm, although this was statistically significant for the late preterm group only (Table 4). Concentrations of plasma uric acid, another marker of metabolic syndrome, were 20.1% higher in the subjects born early and late preterm (Table 4). In addition, plasma albumin and plasma urea concentrations were higher in those born early preterm than in the controls. As for markers of inflammation, the levels of blood leucocytes were higher in those born early preterm (Table 4).

### Associations of perinatal factors

To study whether perinatal conditions that might accompany preterm birth contributed to our findings, we reanalyzed the data by 1) excluding those who were born small for gestational age, 2) excluding those who were born in connection with a pregnancy of multiples, and 3) further adjusting the analyses for maternal gestational diabetes. This did not alter any of the conclusions of the study. We further adjusted the analyses for maternal hypertension in pregnancy. When we adjusted for the variables in model 4, we found that compared with subjects who were not exposed, those who were exposed to maternal hypertension had systolic blood pressures that were 2.6 mm Hg higher and diastolic blood pressures that were 2.0 mm Hg higher. After adjustment for maternal hypertension in addition to other covariates, the differences in systolic and diastolic blood pressures with controls became attenuated to 1.9 (95% CI: −0.3, 4.0) and 1.7 mm Hg (95% CI: 0.1, 3.3), respectively, for early preterm and 0.5 mm Hg (95% CI: −1.2, 2.3) and 0.02 mm Hg (95% CI: −1.1, 1.6), respectively, for subjects born late preterm; this adjustment also attenuated the differences in fat mass and body fat percentage for those who were born early preterm. Further, we reran the analyses that included birth weight standard deviation score by using a birth weight standard based on serial ultrasound measurements of the fetus (20) instead of the commonly used Finnish standard based on newborn measurements (19). Again, this did not alter our conclusions.

## DISCUSSION

We found that young adults who were born preterm had higher levels of cardiometabolic risk factors and were 2.5 to 4 times more likely to meet the criteria of metabolic syndrome than were their peers who were born at full term. They also had higher levels of emerging cardiometabolic risk factors, which suggests that a range of pathophysiological pathways might jointly underlie these associations. Although the results of previous studies have suggested that those born smallest and most immature have elevated cardiometabolic risk factors, our study shows that these risk factors and the full-blown metabolic syndrome are also present in the much larger group of people who were born less preterm.

### Obesity, body composition, and insulin resistance

A key component underlying many characteristics of metabolic syndrome is obesity, particularly abdominal obesity. We found higher rates of obesity and more central body fat in adults who were born preterm. This differs from the situation in adults who were born severely preterm, who tend to be shorter (26, 27) and have a lower BMI as a result of lower lean body mass and similar fat percentages (9, 10) compared with those born at term. Additionally, in a recent meta-analysis in which they compared 412 adults who born preterm with 538 controls, Parkinson et al. (9) determined that there were no differences in fat percentages; again, this is likely a result of the cases having been born severely preterm, as the mean gestational age of those who were born preterm was 30.6 weeks. Consistent with a previous study in adults with very low birth weight (10), we found higher fasting and 2-hour insulin concentrations among the preterm groups, although the differences in the present study were smaller. A conclusion from these findings is that although the large group of adults who were born less preterm had adverse metabolic characteristics associated with impaired glucose regulation similar to those of adults who were born severely preterm, a main contributing factor to impaired glucose regulation for adults born less preterm might be increased body fat with ectopic distribution, which includes increased hepatic fat accumulation; whereas for the smallest preterm individuals, a main contributing factor to impaired glucose regulation may be low muscle mass.

Our subjects were relatively young. As expected, few of them fulfilled the criteria for type 2 diabetes mellitus or impaired glucose tolerance. Studies in older adults have suggested that higher rates of type 2 diabetes mellitus occur in adults who were born preterm (27–30); however, such studies are based on national registers or self-reporting and, thus, involve a degree of uncertainty. Our study, on the other hand, presents direct evidence that abnormal metabolic characteristics are already present in young adult life.

### Blood pressure

Our findings are consistent with a dose-response relationship between a shorter length of gestation and higher blood pressure, a finding that has been supported by population-based studies (14, 31) as well as studies of adults who were born preterm with a very low birth weight (5, 6, 10, 32). High blood pressure is strongly associated with cardiovascular mortality (33, 34) and, globally, is a leading risk factor of death and disease (35). Small differences can be important; for example, although we found a difference of 2.5 mm Hg in diastolic blood pressure between the early preterm group and controls, a difference of 2 mm Hg is associated with a 15% reduction in the risk of stroke (36).

### Lipid profile

Previous findings that concern the association between preterm birth and serum lipid levels in adulthood have been inconsistent (9, 10, 37, 38). We found that women who were born early preterm had lower levels of HDL-C and its precursor apolipoprotein A1 than did women who were born at term. The difference of 11.4% that we found in HDL-C level corresponds to approximately 0.21 mmol/L. A reduction of 0.26 mmol/L has been associated with a 10% increase in the risk of coronary heart disease (39).

### Other biochemical markers of metabolic syndrome

In addition to the established components of metabolic syndrome, we found alterations in a wide range of biomarkers that reflect different underlying pathophysiological pathways. These include uric acid, the concentrations of which were more than 20% higher in both the early and late preterm groups than in the controls. Levels of liver transaminases were also higher in adults who were born preterm, to such an extent that participants with a moderate or high fatty liver index (a proposed marker of nonalcoholic fatty liver disease) were almost exclusively born preterm. We are unaware of any previous reports on uric acid or liver enzymes in adults who were born preterm. Uric acid stimulates oxidative stress, endothelial dysfunction, inflammation, and vasoconstriction and is a strong predictor of type 2 diabetes mellitus and cardiovascular disease, independent of other metabolic syndrome components (40). Liver transaminases and nonalcoholic fatty liver disease also predict these disorders, although the literature is less consistent with regard to the extent that they are independent indicators of pathology rather than general markers of metabolic syndrome (41, 42).

### Limitations and strengths of the study

The main strength of the present study is the study population, which was chosen to include the whole range of preterm births in a specific geographic area. Another strength is the comprehensive measurements of conventional and emerging cardiometabolic risk factors. As to limitations, participation bias cannot be excluded, although a detailed nonparticipant analysis did not raise any concern for such bias. In particular, in the proportion of participants who had undergone an examination at 16 years of age, there was no indication that participants who were born preterm and who had elevated cardiometabolic risk factors during the study period would have been overrepresented in the preterm groups. Although we adjusted for several key confounders, residual confounding remains a possibility. In addition, collider stratification bias is possible after adjustment for intermediate factors, such as BMI, physical activity level, and smoking status, in the regression models; this is unlikely to have any significant effect, as these adjustments had a negligible effect on the results. There was some difference in the proportion of the late preterm and control groups recruited through the NFBC or FMBR; therefore, we adjusted for the recruitment cohort. We had no data to distinguish between spontaneous and medically indicated preterm birth. Instead, we relied on proxy measures, such as being small for gestational age or maternal hypertension in pregnancy, that, nevertheless, are likely to cover a major proportion of indicated preterm deliveries. Moreover, although we had sufficient power for most outcomes, power was limited for more rare outcomes, such as moderate or high fatty liver index.

### Conclusions

We found that young adults who were born preterm had elevated levels of conventional and emerging cardiometabolic risk factors associated with metabolic syndrome, as well as a 2.5 to 4 times greater risk of full-blown syndrome than those born at term. These risks were also present in the large group of young adults who were born late preterm, which is consistent with a dose-response relationship between the degree of prematurity and metabolic syndrome. Our results call for the targeted promotion of a healthy lifestyle and vigilance in the early detection of metabolic syndrome in the over 10% of people born preterm.

## Supplementary Material

Web Material
