# Supplementary material for: Cardiometabolic Risk Factors in Young Adults Who Were Born Preterm
Source: Am J Epidemiol. 2015 May 5;181(11):861–73. doi: 10.1093/aje/kwu443 (PMC4445394; doi:10.1093/aje/kwu443)
Supplement: Web Material [file supp_181_11_861__index.html]

Cardiometabolic Risk Factors in Young Adults Who Were Born Preterm — Web Material 

# Cardiometabolic Risk Factors in Young Adults Who Were Born Preterm

## Web Material

Web Material

- Web Material - Pdf file
