# Supplementary material for: Cardiometabolic Risk Factors in Young Adults Who Were Born Preterm
Source: Am J Epidemiol. 2015 May 5;181(11):861–73. doi: 10.1093/aje/kwu443 (PMC4445394; doi:10.1093/aje/kwu443)
Supplement: Web Material [file supp_kwu443_kwu443supp.pdf]

Web Material

Cardiometabolic Risk Factors in Young Adults Born Preterm

Web Table 1. Confirmation of Lengths of Gestation of the Subjects, Northern Finland, 2009-2011.

Web Table 2. Characteristics of the participants and nonparticipants according to Northern Finland Birth Cohort (NFBC) 1986 and Finnish Medical Birth Register data, Northern Finland 1985-1986 and 2001-2002.

Web Appendix: Laboratory Analyses

Web Table 3. Measured and Calculated Cardiometabolic Markers Grouped According to Pathophysiological Pathways, Northern Finland, 2009-2011.

Web Table 4. Geometric means (SD) for controls and mean differences (95% CIs) in plasma lipids in young women and men born preterm compared with their controls.

Web Table 1. Confirmation of Lengths of Gestation of the Subjects, Northern Finland, 2009-2011.

Gestational age according to NFBC/FMBR data	Gestational age according to medical records		
	Early preterm	Late preterm	Controls
<34 wk.	145	3	4
34-36 wk	4	238	23
≥37 wk.	0	7	353
Total n in GA group*	149	248	380

Abbreviation: FBMR, Finnish Medical Birth Register; GA, gestational age; NFBC, Northern Finland Birth Cohort.
*Gestational age was missing in 2 subjects after conformation of the gestational age.

Web Table 2. Characteristics of the participants and nonparticipants according to Northern Finland Birth Cohort (NFBC) 1986 and Finnish Medical Birth Register data, Northern Finland 1985-1986 and 2001-2002.

Characteristics	Early preterm		P Value	Late preterm		P Value	Controls		P Value
	Study Participants	Study Non-participants		Study Participants	Study Non-participants		Study Participants	Study Non-participants	
No. of subjects	134	188		242	371		344	613	
Male, No. (%)	65 (48.5)	78 (41.5)	0.08	120 (49.6)	226 (60.9)	0.006	169 (49.1)	354 (57.7)	0.01
Mother's age, mean (SD), y	28.9 (5.4)	28.1 (5.6)	0.48	28.4 (6.0)	28.1 (6.1)	0.51	28.0 (5.5)	27.7 (5.4)	0.77
Maternal hypertension in pregnancy, No. (%)	24 (27.6)	21 (16.8)	0.06	16 (11.9)	27 (14.4)	0.52	2 (1.5)	7 (2.9)	0.39
Maternal smoking during pregnancy, No.(%)	19 (14.2)	33 (17.6)	0.42	61 (25.2)	105 (28.3)	0.40	68 (19.8)	150 (24.5)	0.10
Multiple pregnancy, No.(%)	32 (23.9)	35 (18.6)	0.25	33 (13.6)	68 (18.3)	0.13	4 (1.2)	9 (1.5)	0.70
Primiparous, No. (%)	53 (39.6)	81 (43.1)	0.53	105 (43.4)	128 (34.5)	0.03	106 (30.8)	202 (33.0)	0.50
Maternity hospital, No. (%)			0.05			0.93			0.87
Primary or secondary	101 (75.4)	158 (84.0)		184 (76.0)	281 (75.7)		272 (79.1)	482 (78.6)	
Tertiary	33 (24.6)	30 (16.0)		58 (24.0)	90 (24.3)		72 (20.9)	131 (21.4)	
Gestational age at birth, mean (SD), weeks	31.8 (1.6)	31.7 (1.8)	0.38	35.5 (0.9)	35.5 (0.7)	0.29	39.6 (1.3)	39.6 (1.2)	0.13
Birth weight, mean (SD), g	1780 (486)	1798 (554)	0.38	2675 (513)	2697 (538)	0.93	3568 (479)	3629 (488)	0.46
Standard deviation score for birth weight (SD)	-0.75 (1.4)	-0.36 (2.5)	0.16	-0.63 (1.3)	-0.61 (1.3)	0.63	-0.03 (1.0)	0.08 (1.0)	0.51
Birth weight standard deviation score less than -2.0, No. (%)	22 (16.4)	30 (16.0)	0.91	29 (12.0)	41 (11.1)	0.72	7 (2.0)	13 (2.1)	0.93
Age at 01/01/2009, mean (SD), years	21.4 (1.5)	21.3 (1.5)	0.30	21.7 (1.4)	21.8 (1.5)	0.60	22.0 (1.4)	22.0 (1.4)	0.86

Web Table 2 (continued).

Characteristics	Early preterm[a]		P Value	Late preterm[a]		P Value	Controls		P Value
	Study Participants	Study Non-participants		Study Participants	Study Non-participants		Study Participants	Study Non-participants	
No. of members of NFBC 1986 at 16 y of age (%)	47 (35.1)	63 (33.5)	0.77	108 (44.6)	184 (49.6)	0.23	208 (60.5)	369 (60.2)	0.94
Participated in 16 y study, No. (%) [b]	40 (85.1)	39 (61.9)	0.007	94 (87.0)	127 (69.0)	0.001	178 (85.6)	255 (69.1)	<0.001
Parental education at 2001–2002, No. (%) [b]			0.29			0.48			0.39
Elementary	2 (5.3)	7 (20.0)		5 (5.6)	8 (6.8)		5 (2.9)	11 (4.2)	
High school	14 (36.8)	11 (31.4)		27 (30.0)	43 (36.8)		75 (44.1)	97 (36.6)	
Intermediate	19 (50.0)	14 (40.0)		42 (46.7)	53 (45.3)		63 (37.1)	116 (43.8)	
University	3 (7.9)	3 (8.6)		16 (7.8)	13 (11.)		27 (15.9)	41 (15.5)	
Body mass index at 16 years, mean (SD) kg/m^2	20.4 (2.7)	21.3 (3.6)	0.27	21.1 (3.1)	21.3 (4.9)	0.70	20.6 (2.8)	21.4 (3.6)	0.02
Systolic blood pressure, mean (SD), mmHg	118.9 (12.8)	118.3 (11.8)	0.85	116.7 (13.7)	117.5 (13.7)	0.72	115.3 (12.3)	115.4 (13.7)	0.95
Diastolic blood pressure, mean (SD), mmHg	70.5 (8.0)	69.3 (7.5)	0.56	67.9 (7.2)	67.6 (8.1)	0.81	67.8 (7.2)	67.1 (8.1)	0.38
Fasting plasma glucose, mean (SD), mmol/l	5.1 (1.08)	5.2 (1.13)	0.64	5.2 (1.06)	5.1 (1.12)	0.68	5.1(1.08)	5.2 (1.09)	0.19
Fasting serum insulin, mean (SD), mmol/l	10.0 (1.47)	10.8 (1.65)	0.23	9.8 (1.54)	9.9 (1.69)	0.89	8.9 (1.51)	9.9 (1.53)	0.01
HOMA-IR, mean (SD)	1.3 (1.63)	1.3 (1.75)	0.89	1.3 (1.46)	1.4 (1.62)	0.54	1.1(1.52)	1.3 (1.52)	0.006
Total cholesterol, mean (SD), mmol/l	4.4 (1.20)	4.4 (1.15)	0.72	4.2 (1.22)	4.2 (1.20)	0.76	4.2 (1.17)	4.2 (1.22)	0.72
HDL cholesterol, mean (SD), mmol/l	1.4 (1.27)	1.4 (1.23)	0.78	1.3 (1.25)	1.4 (1.24)	0.34	1.4 (1.23)	1.4 (1.22)	0.87
LDL cholesterol, mean (SD), mmol/l	2.3 (1.36)	2.2 (1.27)	0.62	2.2 (1.28)	2.2 (1.32)	0.97	2.2 (1.24)	2.2 (1.30)	0.99
Triglycerides, mean (SD), mmol/l	0.8 (1.65)	0.7 (1.54)	0.39	0.8 (1.56)	0.8 (1.54)	0.61	0.8 (1.49)	0.7 (1.50)	0.61
Apolipoprotein A1, mean (SD), g/l	1.4 (1.13)	1.4 (1.16)	0.16	1.3 (1.17)	1.4 (1.15)	0.11	1.4 (1.16)	1.3 (1.15)	0.55
Apolipoprotein B, mean (SD) g/l	0.7 (1.34)	0.7 (1.27)	0.97	0.7 (1.25)	0.7 (1.31)	0.81	0.7 (1.22)	0.6 (1.29)	0.66
Physical disability at 16 years, No. (%)†	0 (0.0)	8 (4.3)	0.02	1 (0.4)	6 (1.6)	0.17	1 (0.3)	7 (1.1)	0.17

[a]Means other than height are geometric means and strandard deviations (SD). The geometric mean is the nth root of the product of n values. Geometric standard deviations correspond to the percentage increase in a variable corresponding to one standard deviation unit change in the logarithm of the variable.
[b] % of the NFBC members.

WEB APPENDIX: LABORATORY ANALYSES

Plasma glucose (fasting and 2-hour), total cholesterol (TC), high- and low-density lipoprotein cholesterol (HDL-C and LDL-C), triglycerides (TGs), uric acid, alanine aminotransferase (ALT), aspartate transaminase (AST), gamma glutamate, albumin and urea were analyzed by using an Advia 2400 automatic chemical analyzer (Siemens Diagnostics, Terrytown, NY, USA) and blood leucocytes were counted in an automatic electronic cell counter (Abbott CELL-DYN Saphire, Abbott Diagnostics, USA) at Oulu University Hospital laboratory. Serum concentrations of insulin, lipoprotein (a) (LPa), apolipoprotein A1 (ApoA1), apolipoprotein B (ApoB), high-sensitivity C-reactive protein (hsCRP; values >10 mg/l were excluded from analysis), in the Disease Risk Unit of the National Institute for Health and Welfare. The following methods were used: chemiluminescent microparticle immunoassay (CMIA, Abbott Laboratories, USA) for insulin, an immunoturbidimetric method (Abbott Laboratories, USA) for LPa, ApoA1 and ApoB and a latex immunoassay (Sentinel Diagnostics, Italy) for hsCRP. During the course of the measurements the between-batch coefficient of variation in reference samples was <2.9% for insulin, <1.8% for LPa, <1.9% for ApoA1, <3.2% for ApoB and <9.4% for hsCRP. The limit of quantification for the Architect testosterone assay is 0.08 nmol/l. We used a validated calculator program (available at www.dtu.ox.ac.uk) to define homeostasis model assessment values for insulin resistance (HOMA-IR) from paired fasting glucose and insulin levels.

Web Table 3. Measured and Calculated Cardiometabolic Markers Grouped According to Pathophysiological Pathways, Northern Finland, 2009-2011.

Pathway/Group	Variables	Data Presented in
Metabolic syndrome	Metabolic syndrome	Figure 2, Table 2
Body size and composition	Obesity	Figure 2, Table 2
	Height	Table 3
	Body mass index (BMI)	Table 3
	Waist circumference	Table 3
	Waist-hip ratio	Table 3
	Lean body mass	Table 3
	Fat mass	Table 3
	Percentage body fat	Table 3
Blood pressure	Hypertension	Figure 2, Table 2
	Systolic blood pressure	Table 4
	Diastolic blood pressure	Table 4
Glucose metabolism	Fasting plasma glucose	Table 4
	2-hour plasma glucose	Table 4
	Fasting serum insulin	Table 4
	2-hour serum insulin	Table 4
	Homeostatic model assessment for insulin resistance	Table 4
Lipid profile	Total cholesterol	Web Table 4
	HDL cholesterol	Web Table 4
	LDL cholesterol	Web Table 4
	Triglycerides	Web Table 4
	Lipoprotein (a)	Web Table 4
	Apolipoprotein A1	Web Table 4
	Apolipoprotein B	Web Table 4
Inflammatory markers	High-sensitivity C-reactive protein	Table 4
	Blood leukocytes	Table 4
Fatty liver markers	Fatty liver index >30	Figure 2, Table 2
	Plasma alanine aminotransferase	Table 4
	Plasma aspartate transaminase	Table 4
	Plasma gamma glutamate	Table 4
Other biomarkers	Fasting plasma uric acid	Table 4
	Plasma urea	Table 4
	Plasma albumin	Table 4

Abbreviations: HDL, high-density lipoprotein; LDL, low-density lipoprotein.

Web Table 4. Geometric means [a] (SD) for controls and mean differences (95% CIs) in plasma lipids in young women and men born preterm compared with their controls at term, Northern Finland, 2009-2011.

	Model[b]	Women							N
		Controls	Early preterm			Late preterm			
		Mean (SD)	Mean Difference	95% CI	P Value	Mean Difference	95% CI	P Value	
Total cholesterol, mmol/l		4.7 (1.20)							
	1		-2.3%	-7.0, 2.6	0.34	-0.4%	-4.2, 3.7	0.85	361
	2		-2.6%	-7.6, 2.7	0.33	-1.0%	-5.0, 3.2	0.63	352
	3		-2.3%	-7.0, 2.8	0.37	-0.4%	-4.3, 3.7	0.85	351
	4		-3.1%	-8.2, 2.2	0.25	-1.3%	-5.3, 3.0	0.56	342
	5		-2.2%	-7.3, 3.1	0.41	-0.6%	-4.6, 3.7	0.79	342
HDL cholesterol, mmol/l		1.8 (1.25)							
	1		-11.4%	-16.9, -5.6	<0.001	-3.2%	-8.1, 2.0	0.22	361
	2		-11.9%	-17.8, -5.7	<0.001	-3.6%	-8.6, 1.7	0.18	352
	3		-8.3%	-13.7, -2.5	<0.001	-2.1%	-6.8, 2.9	0.40	351
	4		-8.5%	-14.3, -2.3	<0.001	-2.4%	-7.2, 2.8	0.36	342
	5		-7.2%	-12.9, -1.1	0.02	-1.3%	-6.1, 3.7	0.61	342
LDL cholesterol, mmol/l		2.7 (1.35)							
	1		1.3%	-6.6, 9.8	0.76	0.0%	-6.4, 6.8	1.00	361
	2		1.3%	-7.4, 10.7	0.78	-0.4%	-7.1, 6.8	0.91	352
	3		-0.5%	-8.3, 8.0	0.91	-0.5%	-6.8, 6.3	0.89	351
	4		-1.0%	-9.4, 8.3	0.83	-1.0%	-7.7, 6.1	0.77	342
			-0.4%	-8.9, 9.0	0.94	-0.6%	-7.3, 6.6	0.87	342
Triglycerides, mmol/l		1.0 (1.61)							
	1		1.5%	-11.3, 16.1	0.83	5.1%	-5.8, 17.2	0.37	361
	2		-0.8%	-14.2, 14.7	0.92	1.5%	-9.4, 13.7	0.80	352
	3		-3.1%	-15.1, 10.7	0.65	2.1%	-8.3, 13.7	0.70	351
	4		-6.8%	-19.3, 7.5	0.33	-1.9%	-12.2, 9.7	0.74	342
			-2.6%	-14.9, 11.5	0.70	1.4%	-8.7, 12.7	0.79	342
Lipoprotein (a), mg/l		93.6 (3.28)							
	1		4.9%	-25.5, 47.7	0.78	-6.5%	-29.4, 23.9	0.64	338
	2		0.4%	-30.4, 44.7	0.98	-4.6%	-28.7, 27.6	0.75	329
	3		1.5%	-28.4, 43.9	0.93	-10.3%	-32.6, 19.6	0.46	328
	4		-1.2%	-32.0, 43.5	0.95	-8.3%	-31.7, 23.2	0.57	319
			-1.1%	-32.1, 44.0	0.95	-8.2%	-31.8, 23.6	0.57	319
Apolipoprotein A1, g/l		1.7 (1.21)							
	1		-9.7%	-14.7, -4.5	<0.001	-2.9%	-7.3, 1.7	0.22	347
	2		-11.0%	-16.3, -5.4	<0.001	-4.2%	-8.7, 0.6	0.08	338
	3		-8.2%	-13.3, -2.8	0.003	-2.7%	-7.1, 2.0	0.26	337
	4		-9.6%	-15.0, -3.9	0.001	-4.0%	-8.5, 0.8	0.10	328

			-7.7%	-12.8, -2.3	0.006	-2.5%	-6.7, 2.0	0.27	328
Apolipoprotein B, g/l		0.7 (1.32)							
	1		1.5%	-5.6, 9.2	0.68	-0.1%	-5.9, 6.0	0.98	347
	2		1.2%	-6.6, 9.7	0.77	-0.9%	-7.0, 5.6	0.77	338
	3		-0,6%	-7.6, 6.9	0.86	-0.7%	-6.4, 5.4	0.83	337
	4		-2,1%	-9.6, 6.1	0.61	-1.9%	-7.8, 4.4	0.55	328
			-0,9%	-8.4, 7.3	0.82	-1.0%	-7.0, 5.3	0.74	328

Web Table 4. (Continued.)

	Model[b]	Men							N
		Controls	Early preterm			Late preterm			
		Mean (SD)	Mean Difference	95% CI	P Value	Mean Difference	95% CI	P Value	
Total cholesterol, mmol/l		4.5 (1.22)							
	1		1.3%	-4.3, 7.2	0.66	2.6%	-2.0, 7.5	0.27	349
	2		1.2%	-4.4, 7.2	0.67	0.6%	-4.1, 5.5	0.80	342
	3		1.7%	-4.0, 7.7	0.57	1.6%	-3.1, 6.4	0.52	340
	4		1.4%	-4.3, 7.4	0.65	-1.0%	-5.6, 3.9	0.68	333
HDL cholesterol, mmol/l		1.4 (1.26)							
	1		-1.2%	-7.3, 5.2	0.70	-4.1%	-8.9, 0.9	0.11	349
	2		-2.9%	-8.9, 3.5	0.36	-5.4%	-10.3, -0.3	0.04	342
	3		-1.2%	-7.2, 5.1	0.70	-2.5%	-7.3, 2.6	0.33	340
	4		-2.2%	-8.1, 4.1	0.49	-3.4%	-8.3, 1.7	0.19	333
LDL cholesterol, mmol/l		2.8 (1.37)							
	1		0.0%	-8.8, 9.6	1.00	6.3%	-1.4, 14.6	0.11	349
	2		-0.2%	-9.3, 9.8	0.96	3.4%	-4.4, 11.9	0.40	342
	3		0.6%	-8.3, 10.3	0.90	3.9%	-3.6, 12.0	0.31	340
	4		0.4%	-8.7, 10.5	0.93	0.4%	-7.3, 8.7	0.92	333
Triglycerides, mmol/l		1.9 (1.51)							
	1		6.4%	-6.5, 21.0	0.34	5.9%	-4.7, 17.6	0.28	349
	2		8.3%	-5.1, 23.5	0.24	3.7%	-7.1, 15.6	0.52	342
	3		7.1%	-5.1, 20.9	0.26	0.6%	-8.8, 11.0	0.90	340
	4		7.8%	-4.7, 22.0	0.23	-2.6%	-12.2, 8.0	0.61	333
Lipoprotein (a), mg/l		91.0 (3.19)							
	1		-4.9%	-33.0, 34.8	0.78	2.4%	-23.1, 36.4	0.87	331
	2		-2.0%	-31.9, 40.9	0.91	4.0%	-23.2, 40.7	0.80	325
	3		-5.1%	-33.5, 35.3	0.77	1.7%	-23.9, 35.9	0.91	322
	4		-7.1%	-35.6, 34.1	0.69	-1.1%	-27.3, 34.5	0.94	316
Apolipoprotein A1, g/l		1.4 (1.17)							

	1		-1.7%	-5.8, 2.7	0.45	-2.0%	-5.5, 1.5	0.26	343
	2		-2.7%	-6.9, 1.8	0.23	-3.2%	-6.7, 0.4	0.08	337
	3		-1.7%	-6.0, 2.7	0.44	-1.2%	-4.7, 2.4	0.50	334
	4		-2.1%	-6.4, 2.3	0.34	-2.2%	-5.8, 1.5	0.24	328
Apolipoprotein B, g/l		0.8 (1.31)							
	1		1.8%	-5.9, 10.1	0.66	5.6%	-1.0, 12.6	0.10	343
	2		2.2%	-5.8, 10.8	0.60	3.2%	-3.6, 10.4	0.37	337
	3		2.4%	-5.3, 10.6	0.55	3.4%	-3.0, 10.1	0.30	334
	4		2.2%	-5.6, 10.6	0.59	0.0%	-6.4, 6.9	0.99	328

Abbreviations: CI, confidence interval; SD, standard deviation.

[a] The geometric mean is the nth root of the product of n values. Geometric standard deviation (SD) corresponds to the relative change in a variable corresponding to one standard deviation unit change in the logarithm of the variable. P values are for mean differences between preterm groups and controls.

[b] Multiple regression models are: model 1, adjusted for sex, age and cohort; model 2, adjusted as for model 1 + parental educational attainment, maternal smoking during pregnancy, birth weight SD score, parent's hypertension, diabetes and myocardial infarction /stroke; model 3, adjusted for model 1 + BMI, self-reported physical activity, daily smoking; model 4, adjusted as for model 2 +3; model 5 (women only), adjusted for model 4 + hormonal contraception.
